# Fourier-Domain Optical Coherence Tomography for Monitoring the Lower Tear Meniscus in Dry Eye after Acupuncture Treatment

**DOI:** 10.1155/2015/492150

**Published:** 2015-02-18

**Authors:** Tong Lin, Lan Gong, Xiaoxu Liu, Xiaopeng Ma

**Affiliations:** ^1^Department of Ophthalmology, Eye & ENT Hospital of Fudan University, No. 83 Fenyang Road, Shanghai 200031, China; ^2^Shanghai University of Traditional Chinese Medicine, No. 1200 Cailun Road, Shanghai 201203, China; ^3^Shanghai Research Institute of Acupuncture and Meridian, No. 650 Wanping South Road, Shanghai 200030, China

## Abstract

Dry eye is highly prevalent and has a significant impact on quality of life. Acupuncture was found to be effective to treat dry eye. However, little was known about the effect of acupuncture on different subtypes of dry eye. The objective of this study was to investigate the applicability of tear meniscus assessment by Fourier-domain optical coherence tomography in the evaluation of acupuncture treatment response in dry eye patients and to explore the effect of acupuncture on different subtypes of dry eye compared with artificial tear treatment. A total of 108 dry eye patients were randomized into acupuncture or artificial tear group. Each group was divided into three subgroups including lipid tear deficiency (LTD), Sjögren syndrome dry eye (SSDE), and non-Sjögren syndrome dry eye (Non-SSDE) for data analysis. After 4-week treatment, the low tear meniscus parameters including tear meniscus height (TMH), tear meniscus depth (TMD), and tear meniscus area (TMA) in the acupuncture group increased significantly for the LTD and Non-SSDE subgroups compared with both the baseline and the control groups (all *P* values < 0.05), but not for the SSDE. Acupuncture provided a measurable improvement of the tear meniscus dimensions for the Non-SSDE and LTD patients, but not for the SSDE patients.

## 1. Introduction

Dry eye (DE), a multifactorial disease of the tears and ocular surface, is highly prevalent and has a significant impact on quality of life, affecting 14% to 33% of the adult population worldwide [1]. The integrity of the lacrimal functional unit (LFU) is essential to regulate tear production and clearance and then to sustain a sufficient tear volume of appropriate composition to preserve and protect the ocular surface [2]. Disturbance or disease of components of the LFU leads to deficiency or compositional change in tears, which is considered to play an important role in the evolution of different forms of dry eye. Aqueous tear-deficient dry eye implies that dry eye is due to a failure of lacrimal tear secretion, which has two major groupings [3]: Sjögren syndrome dry eye (SSDE) and none Sjögren syndrome dry eye (Non-SSDE), while meibomian gland dysfunction (MGD) is a condition of meibomian gland obstruction and is the most common cause of lipid tear deficiency (LTD) [4].

Several treatment protocols are advocated for dry eye disease management including tear supplementation, tear stimulation, anti-inflammatory therapy, and tear retention. Artificial tears, the most frequently used method to treat dry eye, hydrate the eye and provide limited symptomatic relief with preservative-free tears, whereas acupuncture therapy from traditional Chinese medicine appears to be minimally invasive, but it would promote the secretion of tears by the lacrimal gland naturally and independently to provide a sustained relief to dry eye [5–8]. Although multiple studies have supported the use of acupuncture to treat dry eye [5–12], few studies were designed to investigate the effect of acupuncture on different subtypes of dry eye.

Some commonly used measuring methods for dry eye such as Schirmer's test, rose bengal, corneal fluorescein staining (CFS), and tear break-up time (TBUT) are criticized for their invasive nature and low repeatability [13–15]. The need for a noninvasive, quick, and comfortable dry eye diagnostic test led to the development of new method such as optical coherence tomography (OCT), which revealed the potential to analyze tear dynamics and assessment of tear meniscus after application of collagen punctal plugs [16], topical cyclosporine [17], and artificial tears [18]. Furthermore, previous studies have shown Fourier-domain OCT (FD-OCT) tear meniscus assessment to be a valuable clinical tool in the evaluation of tear volume and ocular surface status with acceptable sensitivity, specificity, and repeatability [19–21]. To the best of our knowledge, tear meniscus dimensions after acupuncture treatment have not been examined using FD-OCT. In this study, we investigated the applicability of FD-OCT tear meniscus assessment in the evaluation of acupuncture treatment response in dry eye patients and explored the effect of acupuncture on different subtypes of dry eye compared with artificial tear treatment.

## 2. Methods

### 2.1. Study Design

In a randomized parallel-group interventional clinical study conducted in China, the efficacy of the acupuncture group was compared to the artificial tear group. In order to investigate the effect on different subtypes of dry eye, each group was divided into three subgroups including LTD, SSDE, and Non-SSDE for data analysis. Criteria for classifying subtypes of dry eye are provided in Table 1. The study has been conducted in compliance with the Declaration of Helsinki and was approved by the Ethical Committee of Eye Ear Nose and Throat Hospital of Fudan University, and all participants provided their written informed consent to participate in this study. This single-institution prospective controlled study began on November 1, 2013, and lasted for 5 months.

### 2.2. Subjects

One hundred and eight dry eye patients (212 eyes) diagnosed at the Eye Ear Nose and Throat Hospital of Fudan University were enrolled in this study. All subjects with dry eye met the following criteria: a Schirmer test without anesthesia (S1T) value of less than 10 mm/5 minutes or a TBUT of less than 10 seconds in each eye and one or more moderate dry eye related symptom (dryness, foreign body sensation, burning, asthenopia, blurred vision, and sensation of stabbing pain). LTD patients showed the presence of meibomian gland dysfunction including meibomian gland dropout by transillumination through the tarsus, no or poor meibum expression in response to digital pressure, and meibomian gland orifice obstruction [22, 23]. SSDE participants fulfilled the American-European Consensus Group classification criteria [24] and showed at least one of the following autoantibodies in serum: antinuclear antibody, rheumatoid factor, anti-SS-A (Ro), or anti-SS-B (La). Subjects were excluded if they had prior corneal transplantation surgery, prior surgery of the lacrimal system, a history of contact lens wear, use of topical medications other than preservative-free artificial tears, or chronic use of systemic medications known to reduce tear production. In addition, subjects were excluded if they had active ocular surface or corneal inflammation or infection, eyelid disorders causing exposure of the ocular surface, or clinically significant conjunctivochalasis. Subjects were also excluded if they had involved other studies within a month.

### 2.3. Study Treatment

The study period was 6 weeks, and subjects who met the inclusion criteria at the end of the 2-week washout continued with the 4-week treatment period. During the run-in period, in order to minimize the therapeutic effect, the subjects were asked to stop any treatments for dry eye for two weeks. All subjects were randomized into the acupuncture group and artificial tear group according to a permuted block method in a 1 : 1 ratio. The acupuncture group participants were offered a total of twelve acupuncture treatment sessions (three times per week for 4 weeks). Acupuncture was performed by the same licensed Chinese medicine physician with more than 5 years of clinical experience in acupuncture treatment. The acupoints used in the treatments is according to the traditional Chinese medicine theory and same as that we described in our previous study [8], including Jingming (BL 1), Cuanzhu (BL 2), Yangbai (GB 14), Sizhukong (SJ 23), Taiyang (EXTRA1), Sìbái (ST 2), Hegu (LI 4), Tàichōng (LR 3), Guangming (GB 37), Sanyinjiao (SP 6), and Fengchi (GB 20). For the periocular points including BL 1, BL 2, GB 14, SJ 23, EXTRA 1, and ST 2, the needles were inserted slowly until the patients reported a sore, numb, heavy, or swollen feeling which was called as “de qi,” and acupunctures were retained for 30 minutes. The fingernail-pressure needle insertion was performed in other acupoints, and acupunctures were also retained for 30 minutes. The artificial tear group participants were treated with carboxymethylcellulose sodium eye drops (Allergan, Inc, USA) four times a day for 4 weeks in both eyes. During the clinical study, concomitant use of any treatment which may affect efficacy assessment was prohibited, including treatment drugs for ophthalmic diseases, corticosteroids (local application to the skin other than the eyelids was permitted), lacrimal plugs, punctal occlusion, or moisture chamber spectacles.

### 2.4. Outcome Measurements

Follow-up examinations were performed at the baseline and 4 weeks after the first treatment. All subjects included in this study underwent subjective symptom evaluation, tear meniscus measurements, TBUT, CFS, and S1T.

#### 2.4.1. Subjective Symptom Assessment

Upon entering the study, subjective symptoms were assessed at each examination using the Ocular Surface Disease Index (OSDI), consisted of the bothersome symptoms, visual function, and environmental triggers subscales. The subjective symptoms were scored on a 5-point scale, with a score of 0 indicating least severe and a score of 4 indicating most severe. A derived index score of ≤100 was calculated for each evaluation based on the total number of questions answered, as previously described [25].

#### 2.4.2. Optical Coherence Tomography-Defined Tear Meniscus Parameters

**Figure 1:**
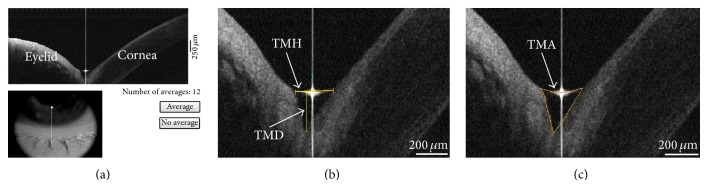
Definition of the tear meniscus parameters. (a) The result of image acquisition by FD-OCT and the measurement of tear meniscus by FD-OCT. The single line scanning mode was selected (scanning line length: 3 mm; scanning direction: 90°–270°). (b) The TMH was the straight-line distance between the upper extreme and the lower extreme of the tear boundary line, whereas TMD was the vertical distance of the interfacing point of the cornea with the lower eyelid to the tear height line. (c) The TMA was the area of the triangle formed by the corneal anterior boundary, anterior boundary of the lower eyelid, and anterior borderline of the tear meniscus.

OCT measurement of the lower tear meniscus was performed as the method described in our previous study [26]. All subjects underwent cross-sectional imaging of the lower tear meniscus prior to instilling drops or measuring any clinical parameters, using an anterior segment OCT system (RTVue-100, Optovue Inc., Freemont, CA, USA). The system takes 26, 000 axial scans per second and has a 5 *μ*m axial resolution and 15 *μ*m transverse resolution. The Cornea-Anterior Module (CAM) software was added to the device for anterior segment imaging. The long-CAM (13 mm wide field) lens adapter was used to take images. The single line scanning mode by anterior segment-wide angle lens was selected (scanning line length: 3 mm; scanning direction: 90°–270°). Optical coherence tomography settings remained constant throughout the experiment for all subjects. Scanning started at the 12 o'clock position of the cornea immediately after the patient blinked. The head was stabilized by an adjustable chin rest and forehead strap. Subjects were instructed to look straight ahead and look at an external target light emitting diode in front of the eye examined. The participant was instructed to blink normally to evenly distribute the tear film and minimize ocular surface dehydration. Just before the measurement, the participant was asked to hold their blink during the acquisition of the scan. Three consecutive scans were performed during each examination with a scanning interval of 3–5 seconds. Tear meniscus height (TMH), tear meniscus depth (TMD), and tear meniscus area (TMA) were determined from the OCT images with RTVue-100 image analysis software. TMH was defined as the straight line distance between the upper extreme and the lower extreme of the tear boundary line; TMD as the vertical distance from the interface of the cornea with the lower eyelid to the tear height line; and TMA as triangular area formed by the corneal anterior boundary, anterior boundary of the lower eyelid, and anterior borderline line of the tear meniscus (Figure 1).

#### 2.4.3. Ocular Surface Clinical Parameters

The ocular surface clinical parameters were all measured by the same observer. Parameters included TBUT, CFS, and S1T. TBUT was measured by instilling fluorescein into the lower fornix with a wetted fluorescein strip (Jingming, Tianjin, China) and the patient was asked to blink several times. Using the cobalt blue filter and slit lamp biomicroscopy, the time required to observe the first area of tear film breakup after a complete blink was recorded as the TBUT. The test was repeated three times and the average of the measurements was calculated. The CFS was graded as previously described on a scale of 0–3 points (none to severe) in each quadrant (total points: 0–12) [27].

S1T was used to measure tear production, by placing a dry Schirmer test strip (Jingming, Tianjin, China) over the outer third of the lower eyelid margin. The distance that the tears traveled along the test strip at 5 minutes was recorded as the S1T score. Potential scores range from 0 to 30 mm, with lower scores indicating reduced function.

#### 2.4.4. Experimental Procedure

The lab settings of the exam room were similar to our previous study [26]. No treatment was given to all the subjects on the visit day. Subjects were tested between 9:00 am and 12:00 am in a consulting room in which central air conditioning and humidifiers controlled the temperature (15°C–25°C) and humidity (30–50%). To avoid reflex tearing, no additional light other than the room light was used in the consulting room. A preliminary anterior segment examination excluded current ocular diseases. Details of the lid margins and lashes, the palpebral and bulbar conjunctival surfaces, the tear film and cornea, and the iris were examined. After a 15 min rest, patients answered an OSDI questionnaire and then were evaluated by OCT examination. Ten minutes after OCT scanning, TBUT was determined and CFS was performed. After a 30 min rest, S1T was conducted.

### 2.5. Data Analysis

All statistical analyses were performed with SPSS for Windows (Ver. 18.0, SPSS, Inc., Chicago, IL, USA). Data are expressed as the mean ± standard deviation (SD). Two-sided pairwise comparisons were conducted (*P* < 0.05). Kruskal-Wallis test was used to determine differences between the acupuncture group and artificial tear group. Two-way ANOVA was used to determine differences among the different subgroups in the acupuncture group and artificial tear group. Statistical significance level was set at 0.05.

## 3. Result

### 3.1. Study Population

**Figure 2:**
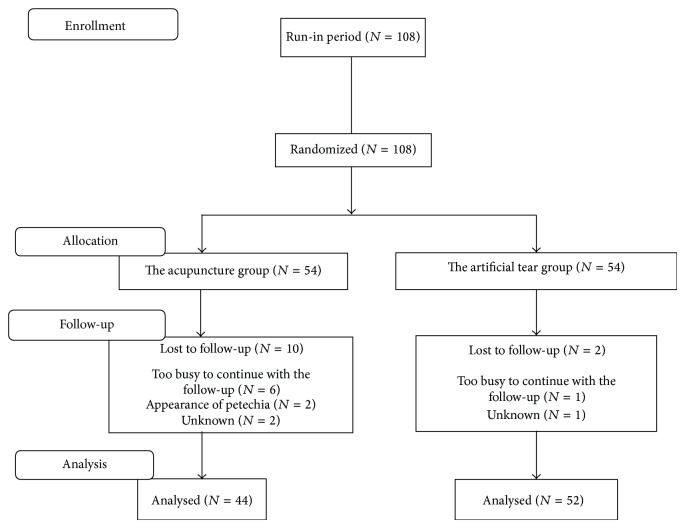
Flow diagram showing the design of the randomized controlled study. A total of 108 patients were enrolled and randomized into the acupuncture group (*n* = 54) or the artificial tear group (*n* = 54). The data of 44 in the the acupuncture group and 52 in the artificial tear group were available for analysis.

Ninety-six patients (188 eyes) were included in the final Full Analysis Set (FAS) comprising 44 patients (86 eyes) in the acupuncture group and 52 patients (102 eyes) in the control group, because 12 patients were lost for the complete follow-up (10 in the acupuncture group and 2 in the control group, Figure 2). The demographic information for the two groups and their subgroups was presented in Table 2. When comparing male-to-female ratio, there was a statistically significant difference between the two groups for the LTD (*P* = 0.0276) and SSDE (*P* = 0.0385) patients. There was no significant difference in mean age and duration of dry eye when the acupuncture group was compared with the control group for all the subgroups (All *P* values > 0.05).

### 3.2. Baseline of OCT-Defined Tear Meniscus Variables and Clinical Parameters

For each group, the mean and standarddeviation values at the baseline for OCT-defined tear meniscus variables and clinical parameters of different dry eye subtypes are summarized in Table 3. There were no significant differences between the two groups for all the parameters except for the S1T value (only for the LTD subgroup, *P* = 0.0242).

### 3.3. Mean Changes of OCT-Defined Tear Meniscus Variables

**Figure 3:**
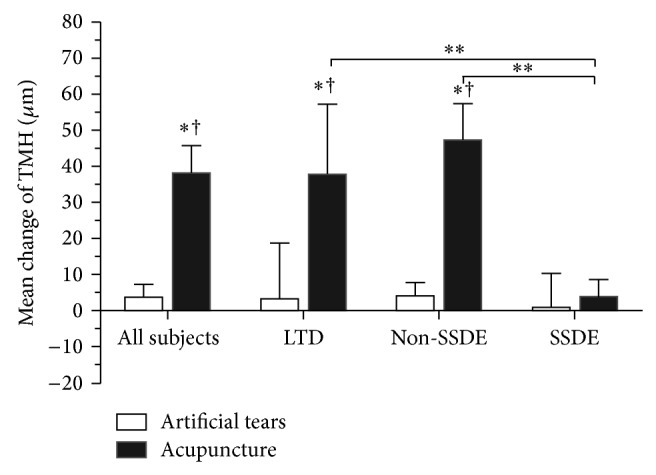
Mean change of tear meniscus height (TMH) from baseline after 4 weeks of treatment for all subjects and each subgroup in acupuncture and artificial tear groups. ^†^significant change from the baseline (*P* value < 0.05). ^**^significance compared to artificial tear group (*P* value < 0.05). ^**^significance between subtypes indicated (*P* value < 0.05). Significant comparisons include acupuncture group VS artificial tear group for all subjects (*P* = 0.0002), LTD (*P* = 0.0036), and Non-SSDE (*P* = 0.0000); LTD VS SSDE (*P* = 0.0330) and Non-SSATD VS SSATD (*P* = 0.0150) in the acupuncture group.

The TMH compared with the baseline in the acupuncture group increased by 38.15 ± 7.52 *μ*m for all subjects (*P* = 0.0000), 37.66 ± 19.46 *μ*m for LTD (*P* = 0.0000), 47.03 ± 10.40 *μ*m for Non-SSDE (*P* = 0.0000), and 3.9 ± 4.90 *μ*m for SSDE (*P* = 0.4586) after 4-week treatment. For the control group, the TMH increased by 3.53 ± 3.69 *μ*m for all subjects, 3.1 ± 12.36 *μ*m for LTD, 3.90 ± 3.88 *μ*m for Non-SSDE, and 0.84 ± 9.44 *μ*m for SSDE (all *P* values > 0.05 compared with the baseline). Significant comparisons were found between groups for all subjects (*P* = 0.0002) and for the subgroups including LTD (*P* = 0.0036) and Non-SSDE (*P* = 0.0000), but not for the SSDE (*P* = 0.2245) (Figure 3).

**Figure 4:**
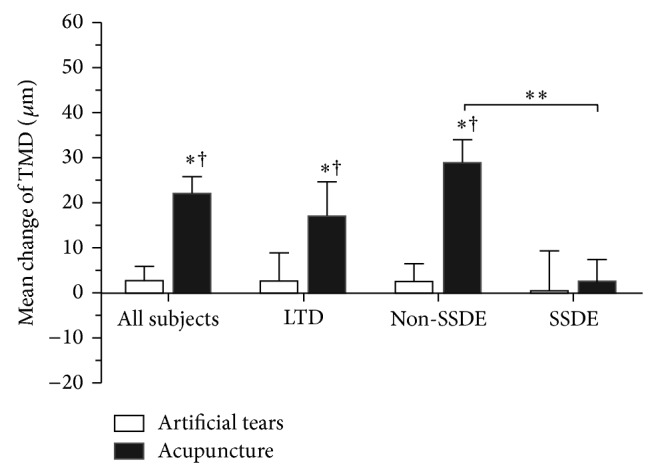
Mean change of tear meniscus depth (TMD) from baseline after 4 weeks of treatment for all subjects and each subgroup in acupuncture and artificial tear groups. ^†^significant change from the baseline (*P* value < 0.05). ^*^significance compared to artificial tear group (*P* value < 0.05). ^**^significance between subtypes indicated (*P* value < 0.05). Significant comparisons include acupuncture group versus artificial tear group for all subjects (*P* = 0.0001), LTD (*P* = 0.0012), and Non-SSDE (*P* = 0.0000); Non-SSDE versus SSDE (*P* = 0.0240) in acupuncture group.

After 4 weeks of therapy, the acupuncture group achieved an improvement of 22.18 ± 3.74 *μ*m (*P* = 0.0000), 17.11 ± 7.71 *μ*m (*P* = 0.0000), 28.92 ± 5.15 *μ*m (*P* = 0.0000), and 2.77 ± 4.89 *μ*m (*P* = 0.5209) of TMD for all subjects and for LTD, Non-SSDE, and SSDE subgroups, respectively. However, the TMD in the control group increased by 2.80 ± 3.10 *μ*m for all subjects, 2.7 ± 7.82 *μ*m for LTD, 2.45 ± 3.98 *μ*m for Non-SSDE, and 0.44 ± 8.92 *μ*m for SSDE (all *P* values > 0.05 compared with the baseline). Significant comparisons were found between groups for all subjects (*P* = 0.0001) and for the subgroups including LTD (*P* = 0.0012) and Non-SSATD (*P* = 0.0000), but not for SSDE (*P* = 0.4507) (Figure 4).

**Figure 5:**
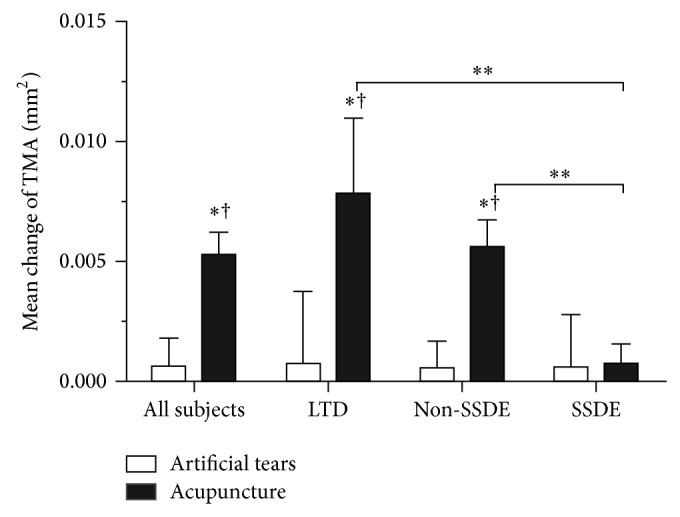
Mean change of tear meniscus area (TMA) from baseline after 4 weeks of treatment for all subjects and each subgroup in acupuncture and artificial tear groups. ^†^significant change from the baseline (*P* value < 0.05). ^*^significance compared to artificial tear group (*P* value < 0.05). ^**^significance between subtypes indicated (*P* value < 0.05). Significant comparisons include acupuncture group VS artificial tear group for all subjects (*P* = 0.0009), LTD (*P* = 0.0256), and Non-SSDE (*P* = 0.0023); LTD VS SSDE (*P* = 0.0190) and Non-SSDE VS SSDE (*P* = 0.0340) in acupuncture group.

The TMA in the acupuncture group increased by 0.0052 ± 0.0009 mm^2^ for all subjects (*P* = 0.0000), 0.0077 ± 0.0031 mm^2^ for LTD (*P* = 0.0000), 0.0055 ± 0.0011 mm^2^ for Non-SSDE (*P* = 0.0000), and 0.0007 ± 0.0008 mm^2^ for SSDE (*P* = 0.3310) after 4-week treatment. For the control group the TMA increased by 0.0005 ± 0.0011 mm^2^ for all subjects, 0.0007 ± 0.0030 mm^2^ for LTD, 0.0005 ± 0.0010 mm^2^ for Non-SSDE, and 0.0005 ± 0.0021 mm^2^ for SSDE (all *P* values > 0.05 compared with the baseline). Significant comparisons were found between groups for all subjects (*P* = 0.0009) and for the subgroups including LTD (*P* = 0.0256) and Non-SSDE (*P* = 0.0023), but not for the SSDE (*P* = 0.6075) (Figure 5).

For theacupuncture group, significant improvements of TMH were found in LTD subgroup (*P* = 0.0330) and Non-SSDE subgroup (*P* = 0.0150) compared with SSDE, respectively (Figure 3). The TMD of Non-SSDE increased significantly compared with SSDE subgroup (*P* = 0.0240, Figure 4). The improvements of TMA were superior in LTD subgroup (*P* = 0.0190) and Non-SSDE subgroup (*P* = 0.0340) compared with SSDE subgroup, respectively (Figure 5).

### 3.4. Subjective Symptoms Analysis

The OSDI score in the acupuncture group decreased significantly by 13.24 ± 13.31 for all subjects, 20.54 ± 10.89 for LTD, 13.16 ± 14.39 for Non-SSDE (all *P* values < 0.05 compared with the baseline), but not for SSDE (*P* = 0.1290) after 4-week treatment. For the control group, the OSDI score showed a significant improvement by 7.17 ± 9.91 for Non-SSDE (*P* = 0.0249), but not for all subjects, LTD, and SSDE. Significant comparisons were found between groups for all subjects (*P* = 0.0016) and for the subgroups including LTD (*P* = 0.0002) and Non-SSDE (*P* = 0.0236), but not for the SSDE (*P* = 0.0910) (Table 4).

### 3.5. Mean Changes of Ocular Surface Clinical Parameters

The ocular surface clinical parameters including the TBUT, CFS, and S1T values were shown to be significantly improved after 4 weeks of treatment for all the subjects in the acupuncture group compared with both the baseline and the control groups (all *P* values < 0.05). The LTD and Non-SSDE patients in the acupuncture group achieved a significant improvement of TBUT and S1T values compared with both the baseline and the control groups (all *P* values < 0.05). However, the SSDE in the acupuncture group did not show significant improvement of all clinical parameters compared with both the baseline and the control groups (all *P* value > 0.05) (Table 4).

### 3.6. Safety

No serious or severe adverse effects occurred in the acupuncture group. Only two cases of petechia were reported in the acupuncture group. Both patients declined further acupuncture treatment and follow-up because of the aesthetic problem caused by the petechia. No adverse events were reported in the artificial tear group.

## 4. Discussion

The current literature on tomographic characterization of the tear menisci suggests that parameters produced by OCT are good quantitative indicators of tear volume [28–32]. Due to its noninvasive and noncontact nature, FD-OCT appears to be very suitable for evaluating tear meniscus variables without any stimulus for possible reflexive tearing. In this study, we demonstrate for the first time the application of FD-OCT to evaluate tear meniscus changes after acupuncture treatment in patients with dry eye. For all the subjects, the acupuncture group achieved significant improvements in the tear meniscus parameters including TMH, TMD, and TMA and clinical parameters including OSDI score, TBUT, CFS, and S1T values compared with the control group. Recent randomized controlled studies in the use of acupuncture in dry eye have been published, with some favourable results [7, 8, 11, 12], which had demonstrated a significant improvement of OSDI score and TBUT, CFS, and S1T values after acupuncture treatment. In this respect, our present study provided more evidences to confirm the effectiveness of acupuncture for dry eye patients.

**Figure 6:**
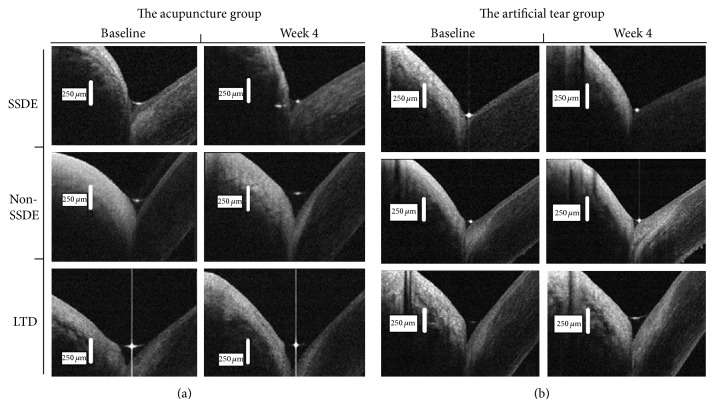
FD-OCT representative images for the lower tear meniscus for each subgroup before and after 4 weeks of acupuncture or artificial tear treatment. (a) Significant improvements of tear meniscus parameters were observed after 4 weeks of acupuncture treatment compared with the control group for the LTD and Non-SSDE subgroups, but not for the SSDE. (b) There were no significant improvements of tear meniscus parameters compared with baseline in the control group for all three subtypes of dry eye.

Artificial tears, the most frequently used method to treat dry eye, were used as the control in this study. Using high-resolution OCT, previous studies demonstrated that the use of artificial tears does not lead to sustained increases in tear volume as blinking returns tear volume to baseline within 30 minutes of artificial tear instillation [18, 33, 34]. In the present study, all measurements had taken at least 8 hours after drop instillation, which may explain why there are no significant improvements of tear meniscus parameters compared with baseline in the control group for all three dry eye subtypes (Figure 6(b)).

Out of China, increasing popularity of acupuncture in many other countries, such as Singapore and South Korea, was shown by the inclusion of conventional physicians as registered acupuncturists in the traditional Chinese medicine board [35]. The recent years witnessed an increase in interest in traditional Chinese medicine especially acupuncture for the dry eye treatment. Despite the encouraging results of recent studies in the treatment of dry eye by acupuncture, little was known about the effect of acupuncture therapy on different subtypes of dry eye. In our study, significant improvements of tear meniscus parameters were observed after 4 weeks of acupuncture treatment compared with the control group for the LTD and Non-SSDE subgroups, but not for the SSDE (Figure 6(a)). The Non-SSDE and LTD in the acupuncture group achieved meanwhile significant improvements of OSDI sore and TBUT and S1T values compared with the control group, but SSDE subgroup did not. These results suggested that acupuncture provided a more effective therapy for LTD and Non-SSDE patients than SSDE patients. Acupuncture treatment is claimed to be able to modulate the autonomic nervous system and immune system, which in turn might regulate lacrimal gland function and increase tear secretion [36, 37], and this action results in a sustained increase of tear volume. Tear meniscus is considered as a reservoir of tears and harbors 75 to 90% of the tear volume on the ocular surface [38]. Therefore, acupuncture group showed superior improvement in tear meniscus variables including TMH, TMD, and TMA for LTD and Non-SSDE patients. However, four weeks of acupuncture therapy alone may not achieve significant recovery for the SSDE. The study from List et al. showed that acupuncture therapy was of a limited value for patients with primary Sjögren syndrome (SS) [39], which was similar to our study. The long-term chronic inflammatory activation within the lacrimal glands causes acinar and ductular cell death and functional impairment [40], which is the main reason for a tear hyposecretion, and it is hard to be improved in SSDE patients. Nevertheless, other protocols such as oral pilocarpine, topical cyclosporine, and punctal occlusion seemed to be effective in improving TMH and the signs and symptoms of dryness in SSDE patients [41–43]. Systemic therapies such as corticosteroids, plaquenil, azathioprine, methotrexate, intravenous (IV) immunoglobulines, and mycophenolate mofetil may also have beneficial effects to SSDE patients [40]. The decision of how to treat the patient depends on the clinical manifestations presented, their severity, and individual considerations [44]. This suggests that there is a need for more than one modality of treatment for SSDE and a combinatorial holistic approach to the management of SSDE may be preferred.

The limitations of this study include the open-label design, the small size of SSDE patients, and the unbalance male-to-female ratio. Future large, population-based studies are warranted to confirm the effect of acupuncture on the treatment of SSDE patients. However, the high resolution and detailed information about the lower tear meniscus obtained by FD-OCT can be valuable clinically in the evaluation of treatment responses. And our present results provide an evidence for clinical application of acupuncture to treat dry eye and select the appropriate treatment strategy for different types of dry eye.

## 5. Conclusion

FD-OCT is capable of monitoring tear meniscus parameters after dry eye treatment with acupuncture. A measurable increase in tear meniscus dimensions was evident after 4 weeks of acupuncture treatment in the Non-SSDE and LTD patients, but not in the SSDE patients.

## Figures and Tables

**Table 1:** Criteria used to define subtypes of dry eye.

Subgroups	OSDI	TBUT <10 Sec	Schirmer <10 mm	MGD	SS
All subjects	>20	+			
LTD	>20	+	−	+	−
SSDE	>20	+	+	−	+
Non-SSDE	>20	+	+	−	−

LTD = lipid tear deficiency; MGD = meibomian gland disease, SS = Sjögren syndrome, ‘‘+” = presence of disease, ‘‘−” = absence of disease; OSDI = Ocular Surface Disease Index; score was determined out of a total of 100; SSDE = Sjögren syndrome dry eye, ‘‘+” = Schirmer <10 mm, ‘‘−” = Schirmer ≧10 mm; TBUT = tear break-up time, ‘‘+” = TBUT <10 sec, whereas ‘‘−” = TBUT ≥10 sec.

**Table 2:** Demographics of all subjects and each subgroup in the artificial tear and acupuncture groups.

Subgroups	*N* (eyes)^†^	*N* (subjects)	Age, mean (y)	Male-to-female ratio	Duration of dry eye, (m)
All subjects					
Artificial tear group	102	52	46	0.44	16
Acupuncture group	86	44	45	0.63	18
*P* value			0.9650	0.6544	0.9130
LTD					
Artificial tear group	19	10	36	0.43	10
Acupuncture group	17	9	41	0.80	12
*P* value			0.8412	0.0276	0.8975
SSDE					
Artificial tear group	18	9	49	0.00	32
Acupuncture group	18	9	44	0.29	30
*P* value			0.7651	0.0385	0.9140
Non-SSDE					
Artificial tear group	65	33	47	0.65	13
Acupuncture group	51	26	46	0.73	16
*P* value			0.9365	0.6801	0.7732

LTD = lipid tear deficiency; SSDE = Sjögren syndrome dry eye; Non-SSDE = non-Sjögren syndrome dry eye. *P* values (<0.05) indicate the statistically significant differences between the acupuncture and artificial tear group. ^†^Four patients (2 in the acupuncture group and 2 in the control group) were enrolled with only one eye for the reason that one of their eyes had been lost because of eye trauma.

**Table 3:** Baseline of tear meniscus variables and clinical parameters of all subjects and each subgroup in artificial tear and acupuncture groups.

Subgroups	OSDI	CFS	S1T (mm)	TBUT (s)	TMH (*μ*m)	TMD (*μ*m)	TMA (mm^2^)
All subjects							
Artificial tear group	46.59 (±15.54)	1.92 (±2.32)	5.42 (±3.73)	4.57 (±1.76)	212.12 (±65.68)	130.90 (±35.65)	0.0175 (±0.0091)
Acupuncture group	51.27 (±20.66)	2.38 (±2.44)	4.70 (±3.26)	4.04 (±2.29)	213.65 (±71.33)	133.71 (±43.29)	0.0181 (±0.0114)
*P* value	0.2083	0.3442	0.3230	0.2027	0.6786	0.3703	0.5489
LTD							
Artificial tear group	48.36 (±15.23)	0.40 (±0.84)	12.00 (±2.10)	4.2 (±1.13)	310.20 (±52.60)	180.8 (±23.12)	0.0307 (±0.0070)
Acupuncture group	42.18 (±20.27)	0.44 (±0.88)	10.22 (±0.44)	3.55 (±1.81)	312.3 (±61.01)	183.5 (±30.83)	0.0314 (±0.0109)
*P* value	0.4597	0.9119	0.0242	0.3602	0.8689	0.6540	0.8169
SSDE							
Artificial tear group	47.85 (±18.33)	6.33 (±1.32)	2.00 (±1.58)	2.22 (±1.98)	155.33 (±29.30)	96.88 (±21.49)	0.0096 (±0.0021)
Acupuncture group	62.08 (±16.52)	6.44 (±1.33)	1.22 (±1.09)	1.88 (±1.16)	154.88 (±49.54)	101.11 (±37.41)	0.0102 (±0.0069)
*P* value	0.1029	0.8614	0.2424	0.6700	0.9636	0.6489	0.8058
Non-SSDE							
Artificial tear group	45.70 (±15.27)	1.18 (±1.10)	4.36 (±1.55)	5.33 (±1.19)	197.87 (±41.97)	125.06 (±24.92)	0.0156 (±0.0065)
Acupuncture group	50.68 (±21.05)	1.65 (±1.26)	4.00 (±1.44)	4.96 (±2.21)	199.78 (±47.31)	127.51 (±35.46)	0.0162 (±0.0093)
*P* value	0.2971	0.1313	0.3617	0.4127	0.6262	0.5424	0.6218

LTD = lipid tear deficiency; SSDE = Sjögren syndrome dry eye; Non-SSDE = non-Sjögren syndrome dry eye; OSDI = Ocular Surface Disease Index; score was determined out of a total of 100; TBUT = tear break-up time; TMH = tear meniscus height; TMH = tear meniscus depth; TMA = tear meniscus area. *P* values (<0.05) indicate the statistically significant differences between the acupuncture and artificial tear group.

**Table 4:** Mean changes of clinical parameters from baseline after 4 weeks of treatment for all subjects and each subgroup in artificial tear and acupuncture groups.

Subgroups	OSDI	CFS	S1T (mm)	TBUT (s)
All subjects				
Artificial tear group	−4.44 (±10.26)	−0.10 (±0.56)	0.00 (±1.58)	0.19 (±0.93)
Acupuncture group	−13.24 (±13.31)^*^	−0.70 (±0.90)^*^	1.75 (±1.46)^*^	1.45 (±1.73)^*^
*P* value	0.0016	0.0001	0.0000	0.0000
LTD				
Artificial tear group	−1.86 (±5.45)	−0.10 (±0.32)	−0.70 (±1.49)	−0.20 (±1.03)
Acupuncture group	−20.54 (±10.89)^*^	−0.33 (±0.71)	2.89 (±1.05)^*^	2.00 (±1.12)^*^
*P* value	0.0002	0.3574	0.0000	0.0003
SSDE				
Artificial tear group	2.69 (±12.15)	0.22 (±1.09)	−0.11 (±1.45)	−0.11 (±0.60)
Acupuncture group	−6.18 (±8.44)	−0.33 (±0.87)	0.56 (±0.53)	0.22 (±0.66)
*P* value	0.0910	0.2494	0.2140	0.2817
Non-SSDE				
Artificial tear group	−7.17 (±9.91)^*^	−0.18 (±0.39)	0.24 (±1.62)	0.39 (±0.93)
Acupuncture group	−13.16 (±14.39)^*^	−0.96 (±0.92)^*^	1.77 (±1.50)^*^	1.69 (±1.98)^*^
*P* value	0.0236	0.0000	0.0005	0.0015

LTD = lipid tear deficiency; SSDE = Sjögren syndrome dry eye; Non-SSDE = non-Sjögren syndrome dry eye; OSDI = Ocular Surface Disease Index; score was determined out of a total of 100; TBUT = tear break-up time. *P* values (<0.05) indicate the statistically significant differences between the acupuncture and artificial tear group. Results indicated with asterisk (∗) denote statistically significant change from the baseline (*P* < 0.05).
